# M2 Muscarinic Receptor Activation Impairs Mitotic Progression and Bipolar Mitotic Spindle Formation in Human Glioblastoma Cell Lines

**DOI:** 10.3390/cells10071727

**Published:** 2021-07-08

**Authors:** Maria Di Bari, Vanessa Tombolillo, Francesco Alessandrini, Claudia Guerriero, Mario Fiore, Italia Anna Asteriti, Emilia Castigli, Miriam Sciaccaluga, Giulia Guarguaglini, Francesca Degrassi, Ada Maria Tata

**Affiliations:** 1 Department of Biology and Biotechnologies Charles Darwin, Sapienza University of Rome, 00185 Rome, Italy; Marina.Dibari@live.it (M.D.B.); vanessa.tombolillo@gmail.com (V.T.); francesco.alessandrini@northwestern.edu (F.A.); Claudia.Guerriero@uniroma1.it (C.G.); 2 Institute of Molecular Biology and Pathology, CNR, 00185 Rome, Italy; mario.fiore@uniroma1.it (M.F.); lia.asteriti@uniroma1.it (I.A.A.); giulia.guarguaglini@uniroma1.it (G.G.); francesca.degrassi@uniroma1.it (F.D.); 3 Department of Experimental Medicine, Section of Physiology and Biochemistry, University of Perugia, 06100 Perugia, Italy; emilia.castigli@gmail.com; 4 Department of Medicine and Surgery, University of Perugia, 06100 Perugia, Italy; miriam.sciaccaluga@unipg.it; 5 Research Centre of Neurobiology Daniel Bovet, 00185 Rome, Italy

**Keywords:** M2 muscarinic receptor, glioblastoma, cell cycle, aberrant mitosis, mitotic spindle

## Abstract

Background: Glioblastoma multiforme (GBM) is characterized by several genetic abnormalities, leading to cell cycle deregulation and abnormal mitosis caused by a defective checkpoint. We previously demonstrated that arecaidine propargyl ester (APE), an orthosteric agonist of M2 muscarinic acetylcholine receptors (mAChRs), arrests the cell cycle of glioblastoma (GB) cells, reducing their survival. The aim of this work was to better characterize the molecular mechanisms responsible for this cell cycle arrest. Methods: The arrest of cell proliferation was evaluated by flow cytometry analysis. Using immunocytochemistry and time-lapse analysis, the percentage of abnormal mitosis and aberrant mitotic spindles were assessed in both cell lines. Western blot analysis was used to evaluate the modulation of Sirtuin2 and acetylated tubulin—factors involved in the control of cell cycle progression. Results: APE treatment caused arrest in the M phase, as indicated by the increase in p-HH3 (ser10)-positive cells. By immunocytochemistry, we found a significant increase in abnormal mitoses and multipolar mitotic spindle formation after APE treatment. Time-lapse analysis confirmed that the APE-treated GB cells were unable to correctly complete the mitosis. The modulated expression of SIRT2 and acetylated tubulin in APE-treated cells provides new insights into the mechanisms of altered mitotic progression in both GB cell lines. Conclusions: Our data show that the M2 agonist increases aberrant mitosis in GB cell lines. These results strengthen the idea of considering M2 acetylcholine receptors a novel promising therapeutic target for the glioblastoma treatment.

## 1. Introduction

Glioblastoma multiforme (GBM) is the most malignant and frequent human brain tumor, representing more than 60% of all brain tumors in adults [1]. Glioblastoma (GB) cells have a highly infiltrative capacity, being able to spread into surrounding brain tissue by using the perivascular space [2]. Despite the great advances made in understanding the molecular alterations that occur in GB, there is not a definitive cure and mortality is still very high. Eighty-eight percent of patients affected by GB succumb to the disease within one to three years [3]. GB is normally treated with surgery, followed by radiotherapy and chemotherapy with temozolomide [4,5]. Therefore, research focused on the development of new therapeutic agents is clinically relevant today.

Muscarinic acetylcholine receptors (mAChRs) are G protein-coupled receptors widely distributed in the nervous system and in several mammalian organs [6]. mAChRs are expressed in several primary and metastatic tumors, such as breast [7], ovarian [8], and lung cancers [9] and in astrocytoma and glioblastoma cells [10,11,12]. In particular, M2 mAChRs appear to be involved in tumor behavior, negatively modulating the proliferation and the migration of cancer cells [13,14,15].

Recently, by in vitro studies, we showed that the selective stimulation of M2 mAChRs by the orthosteric agonist arecaidine propargyl ester (APE) inhibits cell cycle progression and decreases cell survival, both in GB cell lines [14,16] and in GB stem cells [17]. Furthermore, the activation of M2 mAChRs induces apoptosis and oxidative stress, predominantly in p53-mutated glioblastoma cell lines [18].

In recent years, a wide range of clinically employed and experimental anticancer agents arresting the cell cycle and triggering apoptosis have started emerging [19]. Like other solid tumors, GB cells show an aberrant cell cycle and an increased rate of proliferation [20], as well as marked aneuploidy [21], suggesting that one of the causes of chromosomal instability could be determined by deregulated checkpoints.

In the last decade, the phenomenon of mitotic catastrophe has achieved increasing prominence and, in recent years, this mechanism has gained importance as a possible therapeutic target in cancer treatment [22]. In 2012, mitotic catastrophe was defined by the International Nomenclature Committee on Cell Death as an oncosuppressive mechanism that prevents the genomic instability of cells through the induction of mitosis-related cell death or permanent cell cycle arrest [23]. At least three mechanisms of mitotic catastrophe have been described: (I) Activation of the death machinery while the cell is still in mitosis; (II) “mitotic checkpoint adaptation,” i.e., arrested cells enter the interphase without chromosome segregation, and cell death is triggered at the following interphase; (III) development of the senescent phenotype after aberrant mitosis [24,25]. Depending on the status of cell cycle checkpoints, several cytotoxic agents could induce aberrant mitosis/mitotic catastrophe, activating different pathways. Moreover, mitotic catastrophe could also occur after abnormal re-entry of tumor cells into the cell cycle following prolonged growth arrest [26,27].

Previous results have shown that M2 mAChR activation by APE causes the proliferative arrest of U251MG and U87MG cells [16]. Given the ability of APE to increase ROS levels and to cause cytotoxic damage, including chromosomal aberrations [17], in the present work, we investigated in depth the cell cycle progression upon M2 receptor activation in these two cell lines. The data obtained show that M2 agonist treatment causes an arrest in cell cycle progression with an accumulation of cells during pro-metaphase/metaphase transition, resulting in a significant increase in abnormal mitosis and multipolar mitotic spindle formation.

## 2. Materials and Methods

### 2.1. Cell Cultures

U251MG and U87MG cell lines were maintained at 37 °C in an atmosphere with 10% CO_2_. The cells were grown in DMEM (Sigma-Aldrich, St. Louis, MO, USA) plus 10% fetal bovine serum (Sigma-Aldrich, St. Louis, MO, USA), 50 µg/mL of streptomycin, 50 IU/mL of penicillin, 2 mM of glutamine (Sigma-Aldrich, St. Louis, MO, USA), and 1% no essential amino-acids (Sigma-Aldrich, St. Louis, MO, USA).

### 2.2. Pharmacological Treatment

The M2 agonist arecaidine propargyl ester hydrobromide (APE) was used as a preferential agonist of the M2 muscarinic receptor subtype. Pharmacological binding experiments and silencing of the receptor by short interference RNA demonstrated the selectivity of this agonist for the M2 receptor subtype [16,17].

### 2.3. Western Blot Analysis

Cells were homogenized in lysis buffer (Tris-EDTA 10 mM, 0.5% NP40, and NaCl 150 mM) containing protease inhibitor cocktail (Sigma-Aldrich, St. Louis, MO, USA). After protein extraction, the total amount of protein was determined by a Pierce BCA Protein Assay Kit (Thermo Fisher Scientific, Waltham, MA, USA) according to the manufacturer’s protocol. The protein extracts were run on SDS-polyacrilamide gel (SDS-PAGE) and transferred to polyvinylidene difluoride (PVDF) sheets (Merck Millipore, Darmstadt, Germany). Membranes were blocked in 5% non-fat milk powder (Sigma-Aldrich, St. Louis, MO, USA) in PBS containing 0.1% Tween-20, and then incubated with the primary antibodies overnight at 4 °C. The primary antibodies used were SIRT-2 (Santa Cruz Biotechnologies, Dallas, TX, USA) and acetylated alpha-tubulin (Lys 40) (Sigma-Aldrich, St. Louis, MO, USA). The blots were washed three times with PBS + 0.1% Tween-20, then incubated with secondary antibodies conjugated to horseradish-peroxidase for 1 h at room temperature. The immunoreaction was revealed by ECL chemiluminescence reagent (Immunological Science, Rome, Italy). The bands were detected by exposition to Chemidoc (Molecular Imager ChemiDoc XRS + System with Image Lab Software; Biorad, CA, USA).

### 2.4. Flow Cytometry Analysis

The cells were plated at a density of 5 × 10^5^ cells/dish. The day after, the cells, excluding the control samples, were treated either with 100 µM of APE (24 and 48 h of treatment) or with nocodazole (0.2 µg/mL) used as a positive control (24 h treatment). At the end of the treatments, cells were collected by trypsinization and fixed in PBS/ethanol (1:1; *v*/*v*). Samples were then incubated with monoclonal antibodies against phospho-histone H3 (p-HH3) (Ser10) (Merck Millipore, Darmstadt, Germany) for 60 min at room temperature, washed twice with 0.5% Tween-20 in PBS and incubated for 30 min with anti-mouse Alexa fluor 488-conjugated antibody (Invitrogen, Monza, Italy). Samples were washed with PBS and finally stained with 20 µg/mL propidium iodide for 15 min at room temperature. Flow cytometry analysis was performed using a flow cytometer Coulter Epics XL with 488 nm wavelength excitation. For each sample, at least 10^4^ events were measured.

### 2.5. Immunocytochemistry

Cells were fixed with 4% paraformaldehyde for 20 min, permeabilized in PBS containing 0.1% Triton X-100 and incubated in 10% normal goat serum (NGS) for 1 h at room temperature. Coverslips were then incubated overnight at +4 °C with a human CREST antibody (Biolegend, San Diego, CA, USA), diluted 1:50 in PBS containing 0.1% Triton X-100 5% NGS, and then incubated in a Red-X anti-human antibody (1:1000, Jackson Immunoresearch Europe, Cambridge, U.K.) after extensive washing. After a 45 min incubation in a FITC conjugated anti-alpha tubulin antibody (1:100, Sigma-Aldrich, St. Louis, MO, USA), coverslips were washed three times with PBS + 1% BSA and twice with PBS, incubated with Hoechst 33,342 (1:15,000; *v*:*v* in PBS) for 10 min and mounted with glycerol/PBS (3:1; *v*/*v*). In parallel experiments, an anti-acetylated tubulin antibody (1:500, Sigma-Aldrich, St. Louis, MO, USA) and a Red X anti-mouse antibody (1:1000, Jackson Immunoresearch Europe, Cambridge, UK) were used.

### 2.6. M2 Receptor Knock-Down

U251 cells were transfected with a pool of siRNAs selective for human M2 receptors (CHRM2), as previously described [14]. 

The siRNA duplexes were synthesized by Riboxx Life Sciences. The following sequences of siRNAs (Riboxx Life Sciences, Dresden, Germany) used were:

(siRNA1129-1) sense, 5′-AUUUACUACUAAAUCCUCCCCC-3′
antisense 5′-GGGGGAGGAUUUAGUAGUAAAU-3′;
(siRNA1129-2) sense 5′-AUGUAGCCCAUUUCUUCCCCC-3′
antisense 5′-GGGGGAAGAAAUGGGCUACUA-3′;
(siRNA 1129-3) sense 5′-UCCUUUGAGUUUCAGGCUGCCCCC-3′
antisense 5′- GGGGGCAGCCUGAAACUCAAAGGA-3′;
(siRNA 1129-4) sense 5′-AGUUACACCUUGACCUAACCCCC-3′
antisense 5′-GGGGGUUAGGUCAAGGUGUAACU-3′

### 2.7. Time-Lapse Live Cell Imaging

Cells seeded in four-well micro-slides (ibiTreat, cod. 80426, Ibidi, Planegg, Germany) were observed under an Eclipse Ti inverted microscope (Nikon, Tokyo, Japan), using a 40× (Plan Fluor, 0.60 N.A., DIC) objective; during the whole observation, cells were kept in a microscope stage incubator (Basic WJ, Okolab, Pozzuoli, Italy), at 37 °C and 5% CO_2_. DIC images were acquired every 7 min over 72 h using a DS-Qi1Mc camera and NIS-Elements AR 3.22 software (Nikon). Image and movie processing were performed with NIS-Elements HC 4.2. 

### 2.8. Statistical Analysis

Data presented are the average ± SEM obtained from three independent experiments. Statistical analysis was performed by Student’s *t*-tests, Mann–Whitney tests, and one way ANOVAs followed by Tukey’s comparison post-hoc tests. For the evaluation of aberrant mitosis, the ratio between total abnormal metaphases/mitotic cells was calculated. Ten photographic fields for each sample were considered. Each sample was produced in triplicate. Data from time-lapse experiments were statistically analyzed using Mann–Whitney or *χ*^2^ tests, as indicated. The results were considered statistically significant at *p* < 0.05 (*), *p* < 0.01 (**), *p* < 0.001 (***), and *p* < 0.0001 (****).

## 3. Results

### 3.1. M2 Receptor Activation Caused Accumulation of the GB Cell Lines in the M Phase

**Figure 1:**
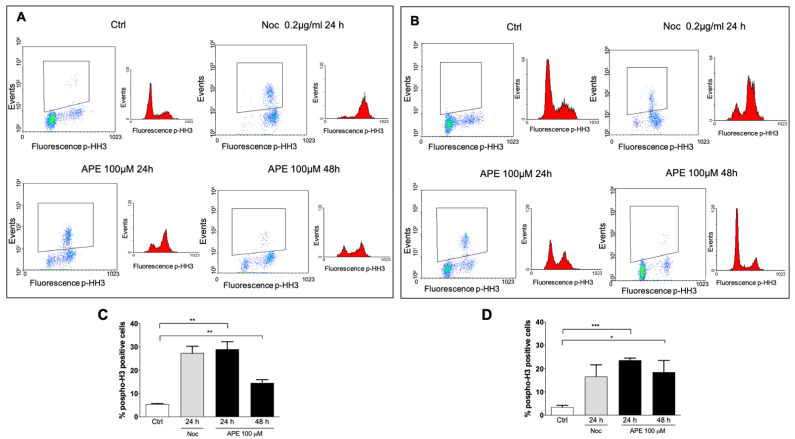
FACS analysis showing the expression of phospho-H3 (ser10) in the U251 (**A**) and U87 (**B**) cell lines. The graphs report the percentage of phospho-H3-positive cells upon treatment with 100 µM of APE or 0.2 µg/mL of nocodazole in the U251 (**C**) and U87 (**D**) cells. Data presented are the average ± SEM of three independent experiments conducted in triplicate. Student’s *t*-test was used to statistically compare the different experimental conditions (*** *p* < 0.001, ** *p* < 0.01, and * *p* < 0.05).

Our previous data indicated that APE induces G2/M arrest in the U251 glioblastoma cell line [16]. In order to verify whether APE was able to induce an arrest in the G2 or M phase, we evaluated—by FACS analyses—the expression of histone H3 phosphorylated at serine 10 (p-HH3), a specific mitotic marker (Figure 1A). Nocodazole, a drug that interferes with microtubule polymerization [28], was used as a positive control. As expected, nocodazole treatment (0.2 µg/mL) caused a significant increase in the percentage of cells positive for p-HH3. Interestingly, APE treatment (100 µM) increased the percentage of p-HH3-positive cells in a comparable manner to nocodazole, at least in terms of 24 h of treatment. As shown in Figure 1C, 30% of treated U251 cells appeared to have accumulated in the M phase. Forty-eight hours after treatment, the percentage of p-HH3-positive cells significantly decreased (18%), albeit remaining higher than untreated cells. 

As previously observed, U87 cells appeared mainly accumulated in the G1 phase after M2 receptor activation [16]. However, an increase in p-HH3-positive cells after APE treatment was observed in U87 cells. In this cell line, only 22% of cells were accumulated in the M phase, mainly 24 h after APE treatment (Figure 1B,D). 

**Figure 2:**
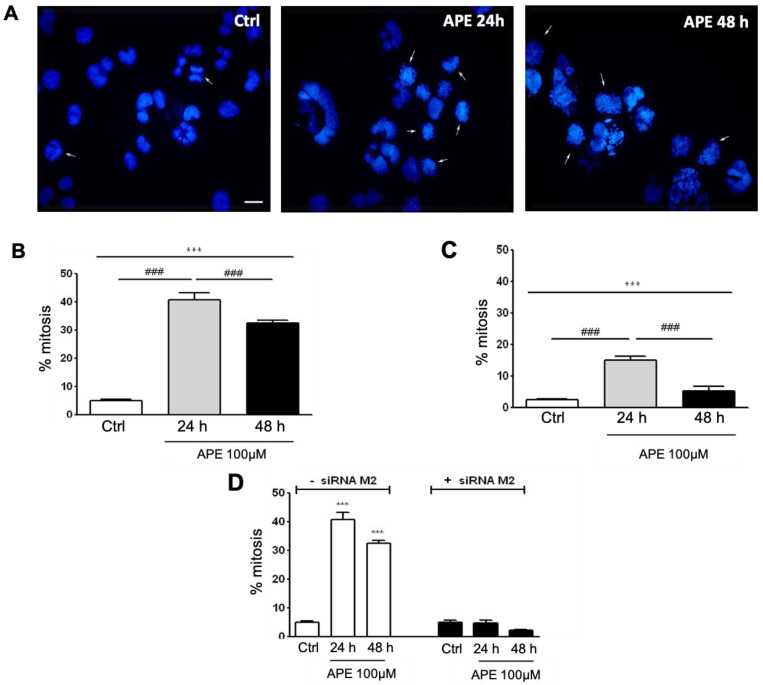
(**A**) Nuclei staining by Hoechst 33,342 in the U251 cells untreated or treated with APE (100 µM) for 24 and 48 h. The arrows indicate the cells in mitosis (bar = 10 µm). The graphs indicate the percentage of cells in mitosis 24 and 48 h after treatment with 100 µM of APE: (**B**) U251 cells and (**C**) U87 cells. (**D**) Percentage of cells in mitosis in the U251 cell line in untreated and APE-treated cells in the presence or absence of M2 receptor knock-down by siRNA transfection. An ANOVA test was used, followed by Tukey’s test, to statistically compare all of the experimental conditions (*** *p* < 0.001; ^###^ *p* < 0.001).

In order to confirm cell accumulation in the M phase, we performed a microscopic analysis on the GB cells after staining with the nuclear dye Hoechst 33342 to assess the frequency of cells in the mitotic stage. Figure 2A is a representative image of the U251 nuclei after APE treatment. Evaluating the nuclei organization and the chromosome distribution, we counted the number of the cells in the mitotic stage at both 24 and 48 h after M2 agonist treatment. As shown in Figure 2B,C, a progressive increase in cells in mitosis was evident after 24 h of APE treatment in both cell lines (U251 and U87). This increase appeared more consistent in U251 than in U87 cells. After longer treatment (48 h), we observed a significant decrease in the percentage of dividing cells.

To verify the direct involvement of the M2 receptor in this phenomenon, we evaluated the percentage of dividing cells in the U251 cell line, treated for 24 h with 100 µM of APE after M2 receptor silencing by siRNA transfection (Figure 2D). After M2 silencing, the percentage of dividing cells in the presence of APE was comparable to that observed in untreated cells, albeit the number of the cells resulted significantly reduced compared to non-transfected cells, most likely as a consequence of the toxicity caused by the transfection.

### 3.2. Analysis of Mitosis Progression

**Figure 3:**
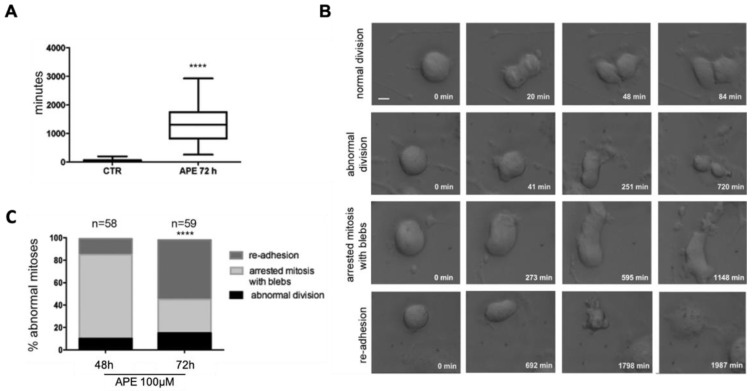
Time-lapse analysis of APE-treated cultures revealing abnormal mitotic phenotypes. The U251 cultures treated with 100 µM of APE were recorded by time-lapse for 72 h upon M2 agonist treatment, and 60–90 mitoses per condition were analyzed. (**A**) Time from mitotic round-up to chromosome segregation (**** *p* < 0.0001; Mann–Whitney test). (**B**) Representative single photograms are shown for a normal mitotic division (first row) and the different observed defects (second to fourth rows); minutes from round-up are indicated. Scale bar: 10 µm. (**C**) Histograms showing the percentage of abnormal mitoses in the APE-treated cultures, divided according to their fate at 48 and 72 h from the beginning of the treatment (**** *p* < 0.0001; Student’s *t*-test comparing 48 vs. 72 h). All mitoses in the control cultures were normal.

**Figure 4:**
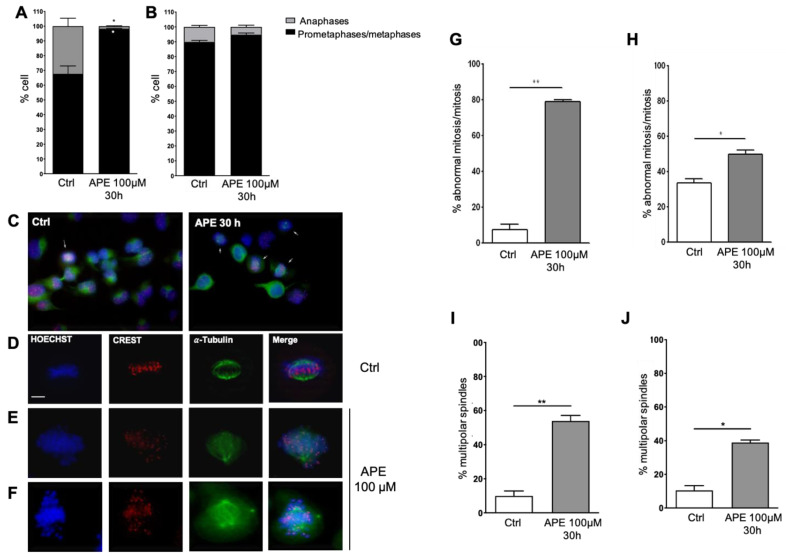
Percentage of pro-metaphases/metaphases and anaphases in the U251 (**A**) and in U87 (**B**) cells after treatment with 100 µM of APE for 30 h. Student’s *t*-test was used to statistically compare the different experimental conditions (* *p* < 0.05). (**C**–**F**) Immunocytochemistry analysis of U251 cells untreated or treated with APE (100 µM) for 30 h. Cells were immunostained with anti-CREST (**red**) and anti-α-tubulin (**green**) antibodies and counterstained with Hoechst 33,258 (**blue**). Scale bars: 5 µm. (**G**,**H**) Percentage of the cells presenting abnormal mitosis in the control condition and after 30 h of treatment with 100 µM of APE, in the U251 and U87 cells, respectively. Student’s *t*-test was used to statistically compare the different experimental conditions (** *p* < 0.01 and * *p* < 0.05). (**I**,**J**) Percentage of the cells with multipolar spindles in the control condition and 30 h after treatment with 100 µM of APE, in the U251 and U87 cells, respectively. Student’s *t*-test was used to statistically compare the different experimental conditions (** *p* < 0.01 and * *p* < 0.05).

In order to directly follow the entrance to the mitotic stage, as well as the progression through mitosis, we videorecorded U251 cells for 72 h after treatment with 100 µM of APE. More than 80% of the cells entered mitosis during videorecording, both in the control and the APE-treated cultures, confirming that no G2 arrest was induced by the treatment. Nonetheless, mitotic entry may be delayed by APE, since we noticed that approximately 30% of the dividing cells entered mitosis between 24 and 72 h after treatment, while the majority (>95%) of control cells progressed to mitosis within the first 24 h (see video in Supplementary Materials). A strong delay/arrest in the pro-metaphase was observed (Figure 3A): While the average time from rounding-up to chromosome segregation onset was approximately 50 min in the control cells, APE-treated mitoses remained for approximately 22 h in a prometaphase-like state and were not able to divide normally. Only 15% of those cells were eventually able to originate two daughter cells through an abnormal division (second row of Figure 3B,C) (see video in Supplementary Materials). The majority of mitoses (>70%) were still arrested in a prometaphase-like state after 48 h and displayed membrane blebbing movements often associated with an elongated shape (third row of Figure 4B,C). At the end of the videorecording (72 h), more than 50% of the analyzed cells had exited mitosis without segregating chromosomes (fourth row of Figure 4B,C), suggesting a failure of cell division after the long blebbing phase (see video in Supplementary Materials).

### 3.3. APE-Arrested Glioblastoma Cells during Prometa-Metaphase Transition and Induced Multipolar Mitotic Spindles

To further investigate the effect of APE on mitosis progression, cells in the mitotic stage were classified as pro-metaphase/metaphase or anaphase, considering the position the chromosomes and their organization on the metaphase plate (Figure 4A,B). We chose to analyze the cells 30 h after treatment, since this is a time before the severe cell death observed after 48 h of APE treatment [16,18]. The data obtained indicate that the number of cells in the pro-metaphase/metaphase was higher in the APE-treated cells compared to the untreated cells (control) and was associated with a concomitant decrease in cells in the anaphase. This trend was observed in both cell lines, but it was statistically significant only in the U251 cell line (Figure 4B). Thus, our data indicate that APE consistently arrested glioblastoma cells prior to anaphase chromosome segregation.

The effect of APE treatment on mitotic spindle structure was additionally examined in pro-metaphase/metaphase cells by immunofluorescence microscopy (Figure 4C–F). We used an antibody against alpha-tubulin to detect the microtubules forming the mitotic spindles and an antibody against kinetochore protein (CREST antibody) to visualize the centromere. The presence of bipolar, well-organized, overall fusiform spindles was evident in the untreated cells. Within these spindles, the highly condensed chromosomes appeared to be aligned along the equatorial plate (Figure 4D). On the contrary, strong abnormalities in mitosis were found in the cells treated with APE for 30 h. This treatment caused a significant increase in the percentage of abnormal mitoses in both the U251 and U87 cell lines compared to the control condition, as shown in Figure 4G,H. Thirty hours after APE treatment, some of the chromosomes appeared not aligned at the equatorial plate or with the spindles themselves, being asymmetric and often of multipolar origin (i.e., bipolar spindles) with clustered centrosomes (Figure 4E) or showing several centrosomes and a multipolar organization of chromosomes (Figure 4F). The graphs in Figure 4I,J show a significant increase in multipolar spindles in the APE-treated cells of both glioblastoma cell lines compared to the untreated cells. 

### 3.4. Modulation of SIRT2 and Acetylated Tubulin Expression after APE Treatment

**Figure 5:**
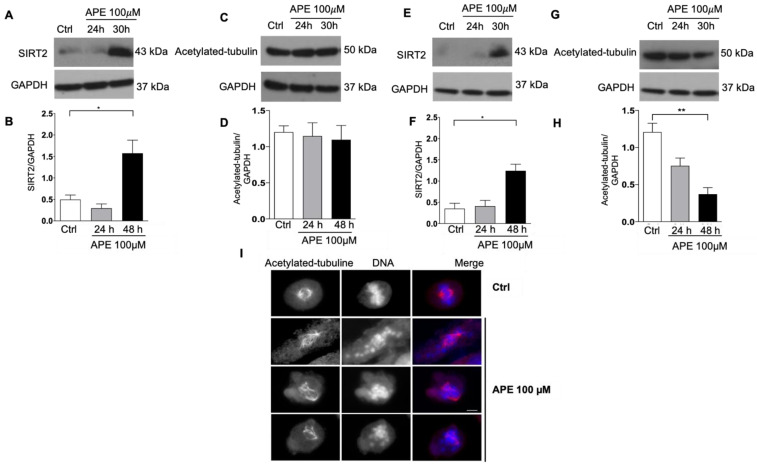
Western blot analysis of SIRT2 and acetylated tubulin expression in U251 and U87 cells treated with APE (100 µM) for 24 and 30 h. Representative blot for SIRT2 (**A**,**E**) and acetylated tubulin (**C**,**G**) in U251 and U87 cells, respectively. GAPDH was used as an internal reference protein. (**B**,**D**,**F**,**H**) Densitometric analysis obtained from three independent experiments in U251 (**B**,**D**) and U87 (**F**,**H**) cells, respectively. Student’s *t*-test was used to statistically compare the different experimental conditions (** *p* < 0.01 and * *p* < 0.05). (**I**) Immunocytochemistry analysis of U251 cells untreated or treated with APE (100) µM for 30 h. Cells were immunostained with an anti-acetylated tubulin (**red**) antibody and counterstained with Hoechst 33,258 (**blue**). Scale bars: 5 µm.

Several pieces of evidence have connected the activity of deacetylase SIRT2 to the control of mitotic progression. Indeed, SIRT2 appears to act in the mitotic checkpoint, blocking chromosome condensation in response to mitotic stress [29]. In light of these findings, we evaluated SIRT2 protein expression by Western blot analysis after APE treatment. As shown in Figure 5A–E, SIRT2 expression significantly increased in the U251 (Figure 5A,B) and U87 cells (Figure 5E,F), particularly 30 h after treatment with 100 µM of APE. Considering that SIRT2 deacetylates tubulin [30], we assessed the expression of acetylated tubulin by Western blot analysis, showing that its levels significantly decreased only in the U87 (Figure 5G,H) cells 24 and 30 h after APE (100 µM) treatment. Apparently, no significant modulation of acetylated tubulin was evident in the U251 cells (Figure 5C,D). Nevertheless, high-resolution immunodetection of acetylated microtubules on mitotic cells demonstrated that the spindle microtubules were extremely abnormal in the U251 treated cells. Meanwhile, the mitotic spindles of the untreated cells consisted of several arrays of acetylated microtubules, and the mitotic spindles in APE-treated cells were characterized by long fibers. These fibers were composed of bundled acetylated microtubules that passed over the chromosomes and did not attach the kinetochores (Figure 5I).

## 4. Discussion

Glioblastoma cells are known to respond to cholinergic stimulation [6,31,32]. The effects of M2 receptor subtype activation on cell growth and survival have been largely characterized using two different glioblastoma cell lines (U251MG and U87MG) in primary cultures obtained from different human biopsies and in glioblastoma cancer stem cells [17]. In particular, we previously demonstrated that M2 receptor activation causes a cell cycle arrest in the G1/S and at G2/M phases in U87MG and U251MG cells, respectively [16]. In addition, cell viability analysis showed that the M2 orthosteric agonist APE causes cytotoxic effects (i.e., ROS production, double-strand DNA breaks, and chromosomes aberrations) and severe apoptosis, especially in p53-mutated cells such as U251MG [18].

Starting from these already described observations, in the present work we investigated in depth the modalities through which the M2 agonist APE triggers the cytostatic effects in glioblastoma cells. 

Since M2 receptor activation arrests cell cycle progression, we analyzed the progression of the mitotic phase. In both glioblastoma cell lines (U251MG and U87MG), we observed an arrest at the mitotic stage, as shown by a significant increase in the percentage of p-HH3 (ser10)-positive cells after M2 agonist treatment. Mitotic arrest was much less evident in the U87MG cell line, in accordance with the higher levels of cells accumulated in the G1 phase than in the G2/M phase, as previously observed in this cell line [16]. Instead, the U251MG cells showed a larger increase in p-HH3-positive cells upon APE treatment, confirming an accumulation of cells in the M phase rather than in the G2 phase, as previously suggested by FACS analysis results [16]. This different behavior may be dependent on the genetic background of two the different glioblastoma cell lines; in fact the presence of wildtype or mutated p53 in U87 and in U251 cells, respectively, may be responsible for the cell accumulation in the different phased of the cell cycle.

Nuclei staining and immunocytochemistry analysis also confirmed that APE caused an accumulation of cells in the M phase. Moreover, a significant increase in abnormal mitosis and an accumulation of cells during pro-metaphase/metaphase transition were observed, accompanied by a reduction in the number of cells in the anaphase. Additionally, the untreated cells formed normal bipolar spindles and the chromosomes appeared aligned along the equatorial plate during the metaphase. Conversely, in the APE-treated cells, asymmetric spindle formation was observed, together with incorrect chromosome alignment along the equatorial plate. In addition, M2 agonist treatment increased the formation of pseudobipolar, tripolar, and multipolar spindles.

To demonstrate the direct involvement of the M2 receptor in abnormal mitosis induction, we silenced *CHRM2* expression by siRNA transfection in the U251 cells, with the cell line showing a higher percentage of abnormal mitosis after M2 agonist treatment. In this condition, the number of abnormal mitoses in the untreated and APE-treated cells was comparable. These results, in accordance with previous studies [16,17], confirm the selectivity of the M2 agonist APE for M2 receptors and show that APE is unable to cause a mitotic arrest in the absence of M2 receptors. 

Several studies have shown that sirtuins, particularly Sirtuin 2 (SIRT2), are among the factors involved in mitotic progression in the normal cell cycle [30]. SIRT2 is an NAD+-dependent histone deacetylase [33]. SIRT2 overexpression has been shown to prolong the M phase and delay mitotic exit [34]. It has been reported that SIRT2 mRNA expression is reduced in approximately 70% of human gliomas [35]. Like SIRT1, SIRT2 confers protection against genotoxic and oxidative stress by reducing cellular ROS levels through the induction of the mitochondrial anti-oxidant enzymes manganese superoxide dismutase (MnSOD) [36] and by blocking chromosome condensation, acting at the level of the mitotic checkpoint [29].

According to the literature data [35], SIRT2 appears to be strongly downregulated, both in U87 and U251 cell lines. Interestingly, APE treatment induced a significant upregulation of SIRT2 expression 30 h after treatment, in line with the increased accumulation of the cells in the M phase. Moreover, SIRT2 activity may contribute to the formation of deacetylated unstable microtubules [37]. In our experiments, we observed a decrease in acetylated tubulin expression, particularly in the U87 cells. This phenomenon may lead to spindle fragmentation, which contributes to the formation of spindle extra poles and abnormal chromosome segregation. Although U251 did not show a significant modulation of acetylated tubulin after M2 agonist treatment, by immunocytochemistry analysis for acetylated tubulin, we observed that in U251 cells treated with APE, the acetylated microtubules passed over the chromosomes and did not attach the kinetochores. Previous data have demonstrated that APE treatment induces oxidative stress, DNA damage, and chromosomal instability, particularly in the U251 cell line [18]. These results suggest that the inability of microtubules to bind chromosomes may also be dependent on the possible alterations that may occur on DNA or other proteins associated with the kinetochores that prevent the interaction between chromosomes and microtubules. Altogether, these alterations may negatively influence chromosomes’ organization and their attachment to microtubules of the mitotic spindle and their inability of divide cells. The dramatically extended mitotic arrest and the highly abnormal phenotypes observed by time-lapse microscopy showed that glioblastoma cells are unable to further proliferate after APE treatment. This suggests that mitotic catastrophic and apoptotic events may occur in subsequent mitosis. 

## 5. Conclusions

Glioblastoma cells are characterized by an aberrant cell cycle [20] and marked aneuploidy [21]. Therefore, mitotic catastrophe induction may be an interesting oncosuppressive mechanism with the aim of inducing mitosis-related cell death or permanent cell cycle arrest [23]. The data obtained in the present work, together with previous data [14,16,17,18,38,39], may allow to hypothesize that the activation of the M2 receptor through selective agonists causes two significant effects in glioblastoma cells: (1) A cytostatic effect, impairing cell proliferation and inducing cell cycle arrest; (2) a cytotoxic effect, increasing apoptosis and cell death. The two effects should be strictly correlated and both contribute to impairment of glioblastoma cell proliferation and survival. 

These results, together with the data obtained in other tumor types (i.e., neuroblastoma, breast cancer, and urothelial cancer) [13,15,40,41], highlight the M2 muscarinic receptor as a new strategic therapeutic target in cancer therapy. Therefore, a special effort to identify new selective ligands able to bind this receptor with more efficacy and to reduce the possible side effects appears clinically relevant and may open new therapeutic perspectives for glioblastoma treatment, as well as for the treatment of other tumors. 

## Supplementary Materials

The following are available online at https://www.mdpi.com/article/10.3390/cells10071727/s1, Video S1: Abnormal cell division in U251 cells.
